# Investigating the dynamics of *S.* Enteritidis serotype spread in breeder poultry company using a whole genome sequencing approach

**DOI:** 10.3389/fvets.2026.1810481

**Published:** 2026-04-14

**Authors:** Qian Wang, Mengze Song, Wei Zhao, Xiaoran Hu, Zhipeng Tang, Longzong Guo

**Affiliations:** 1 Poultry Disease Eradication Research Laboratory, Shandong Agricultural University, Taian, China; 2 Shandong Yisheng Livestock and Poultry Co., Ltd., Yantai, China; 3 Animal Disease Prevention and Control Center of Fushan District, Yantai, Shandong, China

**Keywords:** antimicrobial resistance, breeder farm, *S.* Enteritidis, SNP, WGS

## Abstract

*Salmonella* is a globally prevalent foodborne zoonotic pathogen, with poultry and its products serving as the primary epidemiological reservoirs. As a core upstream component of the poultry industry chain, breeder farms can harbor *Salmonella* that contaminates breeder eggs via vertical transmission or spreads to hatcheries and downstream commercial flocks through horizontal transmission, resulting in cross-generational and cross-regional dissemination of the pathogen. From 2013 to 2022, this study conducted a molecular epidemiological investigation on 252 *Salmonella* isolates from grandparent (GG) and parent (PG) chicken flocks of an integrated breeder poultry enterprise in Shandong Province using whole-genome sequencing (WGS) technology. The results showed that *S.* Enteritidis was the dominant serotype, accounting for 92.85% of all isolates. All *S.* Enteritidis isolates belonged to ST11. The drug-resistant gene among the 252 *Salmonella* with the highest carrying rate was the fluoroquinolone resistance gene *gyrA* (93.68%), followed by the *β-lactam* antibiotic resistance gene *blaTEM-1B* (86.56%), the aminoglycoside resistance genes *aph(3″)-Ib* (81.82%) and *aph(6)-Id* (81.82%), the sulfonamide resistance gene *sul2* (80.63%) and the tetracycline resistance gene *tet(A)* (40.32%). The plasmids with the highest carrying rates are *IncFIB* (90.91%) and *IncFII* (90.91%). Among single-point mutations, *gyrA D87Y* had the highest amino acid substitution rate (80.63%). The drug resistance gene carrier rates were similar between GG and PG isolates. After the comprehensive ban on antibiotics in animal feed implemented in 2020, the carriage rate of the tetracycline resistance gene *tet(A)* decreased significantly from 72 to 26%. SNP distance calculations showed that among the 234 *S.* Enteritidis strains, 163 (69.7%) were homologous to at least one other isolate, with a total of 1,047 clonal transmission events. *S.* Enteritidis underwent extensive clonal transmission across different regions, different generations of culture, and different time periods. This study clarified the transmission patterns and antimicrobial resistance evolution characteristics of *Salmonella* in breeder farms, providing a genomic basis for formulating targeted biosecurity prevention and control strategies as well as antibiotic reduction programs.

## 1 Introduction

*Salmonella* is a major zoonotic foodborne pathogen of global public health importance, accounting for an estimated tens of millions of intestinal infections and hundreds of thousands of deaths annually worldwide (1, 2). Food-producing animals, particularly poultry and livestock, are well-recognized as the primary reservoirs for human *Salmonella* infections (3). With more than 2,600 identified serotypes, *Salmonella* is categorized into typhoidal and non-typhoidal *Salmonella* (NTS) (1). In contrast to *Salmonella Typhi*, which is strictly human-adapted, NTS has a broad host range encompassing poultry, pigs, cattle, rodents, companion animals, and various wildlife species, and is primarily transmitted to humans through the food chain (4, 5). Among NTS, *S.* Enteritidis is one of the most prevalent serovars linked to human salmonellosis, with poultry and its products confirmed as the major epidemiological reservoir (6). Infections caused by *S.* Enteritidis can induce a spectrum of intestinal diseases in both humans and animals, and may progress to severe, life-threatening systemic infections in vulnerable populations (7).

As the upstream segment of the poultry industry, the breeding sector plays a crucial role in *Salmonella* transmission. Poultry can acquire *Salmonella* through multiple pathways during the production and breeding processes. For instance, *Salmonella*-infected breeder chickens can vertically transmit the pathogen to their progeny via eggs (8). Horizontal transmission also occurs through direct contact between animals or indirect exposure to contaminated feed, bedding, water, or aerosols (9). Notably, *S*. Enteritidis can persistently colonize the ovarian tissues of hens, leading to egg contamination via both horizontal and vertical pathways (10, 11). Additionally, *Salmonella* can penetrate the eggshell surface, a process closely associated with fecal excretion by infected hens and the deposition of contaminated eggs (12). Since hens share the cloaca for both egg-laying and defecation, *Salmonella* colonization of the digestive tract can result in eggshell contamination before, during, and after oviposition.

Intensive poultry farming is characterized by high stocking densities and accelerated growth rates, which impede effective hygiene management and facilitate *Salmonella* spread in production environments. Understanding the dynamic transmission of *Salmonella* within large-scale poultry enterprises requires comprehensive analysis of both serotypic and genotypic characteristics (13). Elucidating transmission routes, infection pathways, and genetic relatedness among *Salmonella* strains is critical for developing effective control strategies. With the escalating burden of *Salmonella* infections, traditional molecular typing methods such as pulsed-field gel electrophoresis (PFGE) and multilocus sequence typing (MLST) no longer meet the demands of high-resolution detection. Whole-genome sequencing (WGS) has emerged as a powerful molecular typing tool for epidemiological investigation and traceability, owing to its superior resolution (14). WGS enables rapid generation of complete bacterial genomes, thereby facilitating accurate determination of genetic relationships among strains and inference of their evolutionary history (15, 16).

As the foundational tier of the poultry production pyramid, breeder farms represent a critical control point for pathogen dissemination. Infections established in grandparent (GG) or parent (PG) stock can be amplified through vertical transmission to progeny and horizontal spread within hatcheries, thereby seeding downstream commercial flocks with persistent clones. While numerous studies have documented *Salmonella* prevalence in commercial layers or broilers, longitudinal genomic surveillance within integrated breeder operations remains limited. This study therefore applied WGS to conduct a decade-long (2013–2022) epidemiological investigation of *Salmonella* in a major integrated breeder poultry company in China. We aimed to elucidate the population dynamics, transmission networks, and antimicrobial resistance (AMR) evolution of *Salmonella*, with a specific focus on the dominant serovar Enteritidis, to identify strategic interventions for breaking cycles of infection at this pivotal production stage.

## 2 Materials and methods

### 2.1 Samples and collection sites

For this study, *Salmonella* isolates originating from 1 integrated poultry companies were characterized and compared using whole genome sequencing (WGS). These isolates were collected between 2013 and 2022 from farms in six cities belonging to a poultry farm in Shandong Province, China (Yantai, Weihai, Weifang, Suzhou, Suining, Dongying). The samples collected came from cloacal swabs, feces, environmental samples and dead-in-shell embryos. The types of farms are divided into grandparent (GG) and parent generations (PG).

### 2.2 Whole genome sequencing

The bacteria were sent to Novogene Biology Science and Technology Co., LTD (Tianjin, China) for genome sequencing. Genomic DNA of all 252 isolates was extracted for whole-genome sequencing using the Wizard Genomic DNA Purifcation Kit (Promega) following the manufacture’s instructions. DNA libraries were constructed using NEXTflex Rapid DNA-Seq Kit (Bioo Scientific) following the standard protocols, and then sequenced on the Illumina NovaSeq 6,000 platform (Annoroad). The raw sequence data of these strains was subjected to the quality control procedure by FastQC, then the clean data was assembled using SPAdes v.3.15.4.

### 2.3 Bioinformatics analyses

The assemblies were serotyped by sistr-cmd v1.1.1 (17) and then screened against the *Salmonella* seven-locus multi-locus ST database, Resfinder database (18), PlasmidFinder Database (19), Pointfinder database (20) using Staramr v.2.0.1 (21) to assign STs and detect acquired ARGs, plasmid replicons and point mutations. The results were transformed into a binary table in R v.3.6.0 to analyse the presence/absence of acquired ARG alleles and the prevalence of each gene in different isolates grouped by serotypes or background information. All assemblies were annotated with Prokka v.1.14.6 (22) and subjected to a pangenome analysis with Roary v.3.12.0 (23). The WGS used only short read platform, therefore the complete plasmid studies could not be accomplished.

The full set of 234 genomes was used to generate a core-genome alignment and construct a maximum likelihood phylogenetic tree, using FastTree v.2.1.10 with gtr model. The midpoint rooted phylogenetic tree was annotated in by ggtree and ggtreeExtra. Minimum spanning trees were created based on cgSTs using GrapeTree v1.5.153 (24) with MSTree V2 algorithm. The cgMLST allele profiles were annotated using cgMLST.py v1.2.0 with 3,002 allele genes. Genomic diversity of the isolates within each source was assessed by calculating all pairwise allelic differences (PADs) between any two isolates. When multiple isolates shared the same cgST, one isolate was randomly picked and used for PAD calculation to ameliorate potential sampling biases caused by overrepresentation of closely related isolates.

To obtain more accurate evidence of *Salmonella* transmission, we further calculated the SNP distance using core-gene alignment (core genome size ~3.1 Mb) of 234 genomes with SNP-dists v0.7.0.^1^ Isolate combos differed by ≤5 SNPs were considered as potential transmission case (25). Transmission cases are visualized by ggplot2 within R v.3.6.0.

## 3 Results

### 3.1 Epidemiology and population dynamics of *Salmonella* serotypes

**Figure 1:**
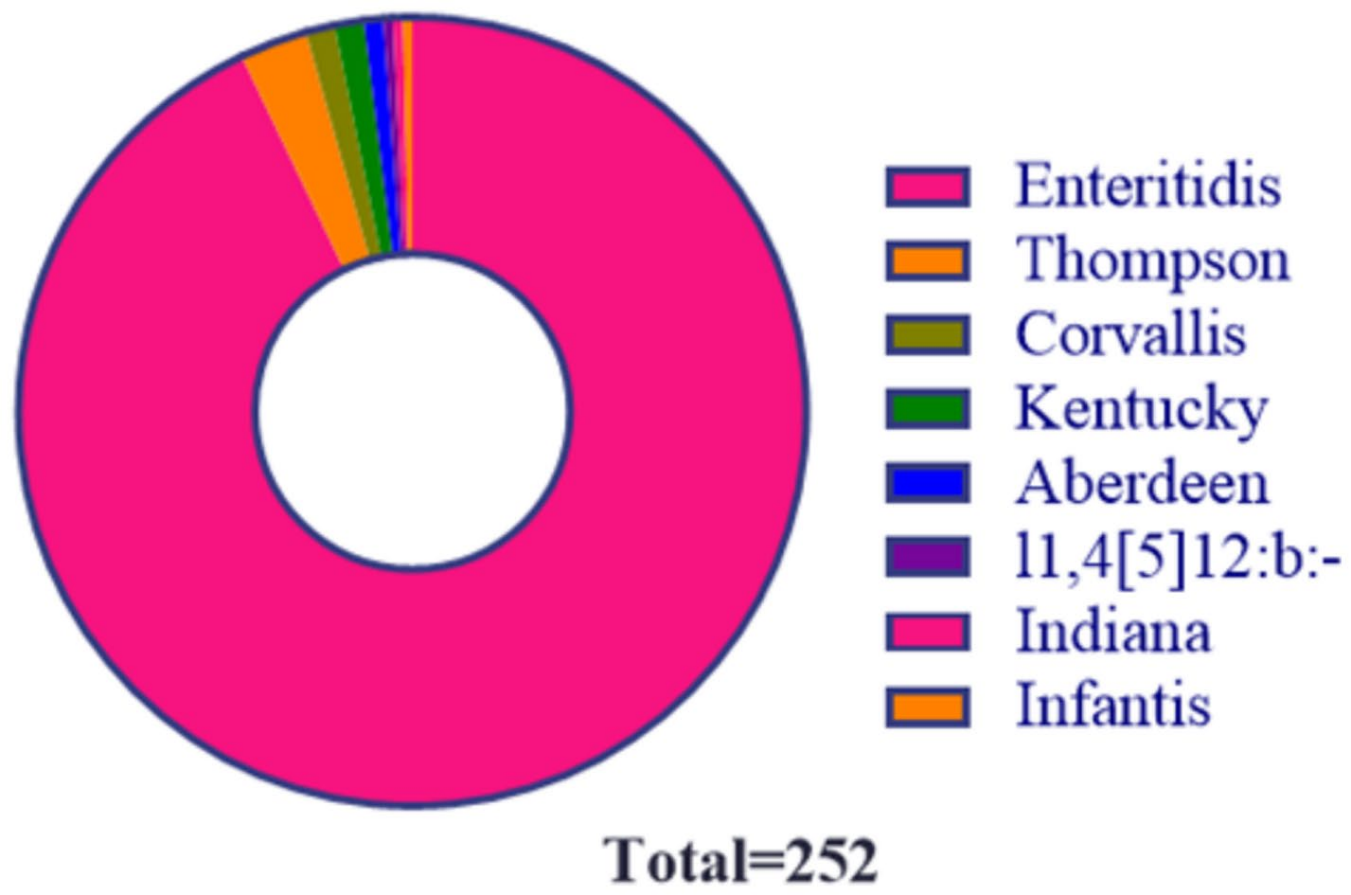
*Salmonella* serotype statistics.

A total of 252 *Salmonella* strains were isolated from chicken farms between 2013 and 2022, including 93 from parent stock (PG) and 159 from grandparent stock (GG) (Table 1). Eight distinct serotypes were identified among the isolates, with notable geographic specificity: *S*. Indiana, *S*. Aberdeen, *S*. Thompson, *S*. I1,4,[5]12:b:-, and *S*. infantis were exclusively isolated from Yantai; S. Kentucky was restricted to Weihai; and *S*. Corvallis was only detected in Suining. In contrast, *S*. Enteritidis was ubiquitous across all surveyed farms. Among the 252 strains, 234 (92.85%) were *S*. Enteritidis, which was the most dominant serotype (Figure 1).

**Table 1:** Frequency and origin of *Salmonella* serotypes in each poultry integration.

Serovar		Yantai	Weihai	Weifang	Suzhou	Suining	Dongying	2013–2020	2021	2022
Enteritidis	PG	29	35	4	3	6	7	47	30	7
	GG	112	38	0	0	0	0	35	50	65
Indiana	PG	0	0	0	0	0	0	0	0	0
	GG	1	0	0	0	0	0	0	1	0
Aberdeen	PG	0	0	0	0	0	0	0	0	0
	GG	2	0	0	0	0	0	0	1	1
Kentucky	PG	0	2	0	0	0	0	2	0	0
	GG	0	1	0	0	0	0	1	0	0
Thompson	PG	2	2	0	0	0	0	4	0	0
	GG	3	0	0	0	0	0	0	3	0
I1,4,[5]12:b:-	PG	0	0	0	0	0	0	0	0	0
	GG	1	0	0	0	0	0	1	0	0
Corvallis	PG	0	0	0	0	3	0	3	0	0
	GG	0	0	0	0	0	0	0	0	0
Infantis	PG	0	0	0	0	0	0	0	0	0
	GG	1	0	0	0	0	0	0	0	1

### 3.2 Drug resistance gene carrier characteristics

**Figure 2:**
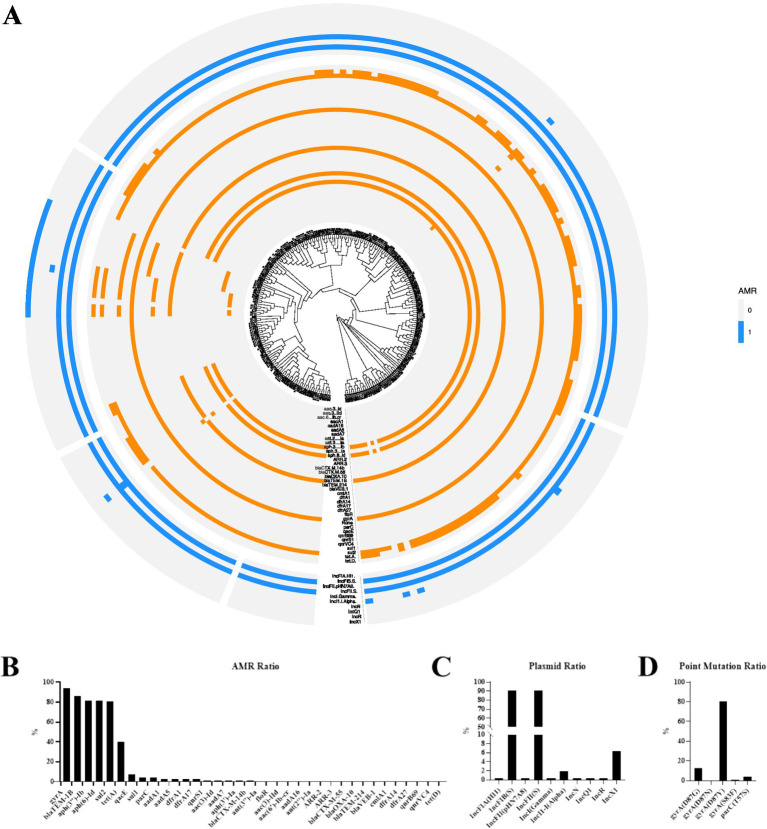
Distribution of drug resistance genes in *Salmonella*. **(A)** Phylogenetic tree; **(B)** Drug resistance spectrum; **(C)** Plasmid ratio; **(D)** Point mutation ratio.

A core-genome phylogenetic tree was constructed for the 252 *S*. Enteritidis strains, and their antimicrobial resistance gene profiles were annotated using the CARD database (Figure 2A). The drug-resistant gene with the highest carrying rate was the fluoroquinolone resistance gene *gyrA* (93.68%), followed by the β-lactam antibiotic resistance gene *blaTEM-1B* (86.56%), the aminoglycoside resistance genes *aph(3″)-Ib* (81.82%) and *aph(6)-Id* (81.82%), the sulfonamide resistance gene sul2 (80.63%) and the tetracycline resistance gene tet(A) (40.32%) (Figure 2B). The plasmids with the highest carrying rates are IncFIB (90.91%) and IncFII (90.91%) (Figure 2C). Quinolone resistance is caused by single-point mutations in the *gyrA* DNA gyrase gene, with the highest amino acid substitution rate at *gyrA* D87Y (80.63%) (Figure 2D). Notably, the resistance gene profile showed strong clustering in the phylogenetic tree, with strains in the same clade sharing similar resistance gene combinations, indicating that resistance traits were predominantly transmitted vertically within clonal lineages.

**Figure 3:**
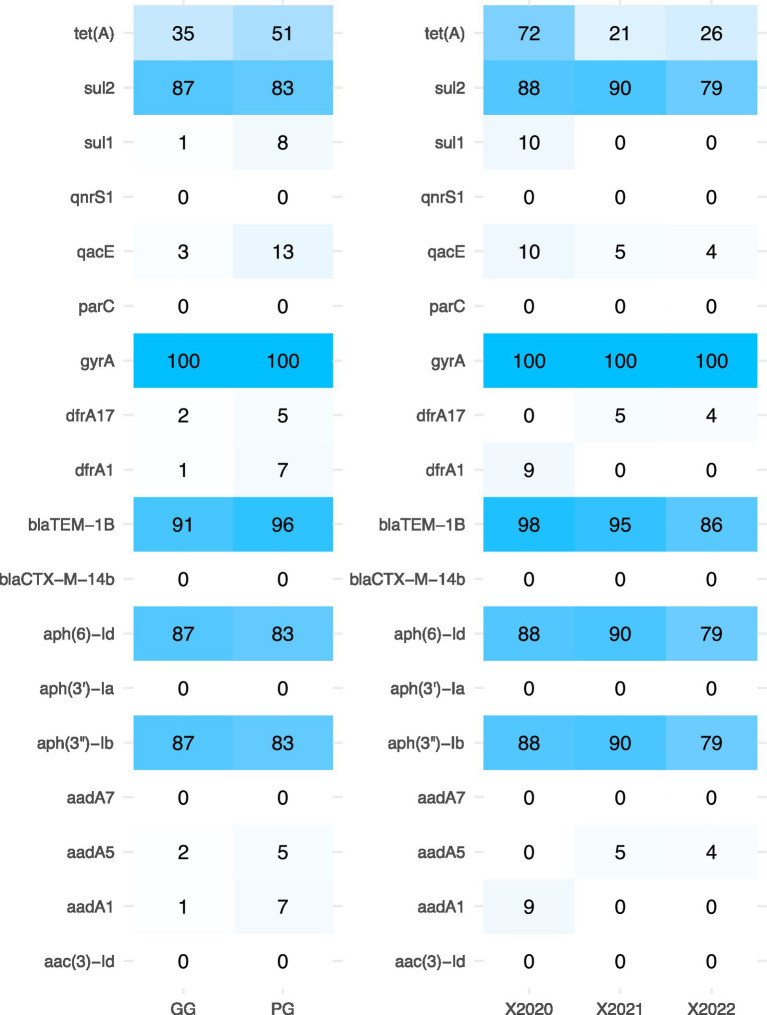
Distribution of drug resistance genes of *S.* Enteritidis.

The 234 *S*. Enteritidis strains were stratified by production houses (GG vs. PG), and their resistance gene profiles were compared. The results showed that there was no significant difference in the drug resistance gene composition of the strains isolated from GG and PG chicken houses, indicating that the drug resistance gene may have been widely spread during several generations of breeding. In 2020, China implemented a comprehensive ban on antibiotic use in animal feed. To evaluate the impact of this policy, resistance gene carriage rates were compared among three groups: 92 strains isolated from 2013 to 2020 (pre-ban period), 85 strains from 2021 (transition period), and 77 strains from 2022 (post-ban period). Statistical analysis revealed no significant differences in the carriage rates of *gyrA*, *blaTEM-1B*, *aph(3")-Ib*, *aph(6)-Id*, and sul2 among the three periods. In contrast, the carriage rate of the tetracycline resistance gene *tet(A)* decreased significantly from 72% (2013–2020) to 21% (2021) and 26% (2022). This reduction is likely attributable to the removal of tetracycline selection pressure following the antibiotic ban, while the persistence of other resistance genes may reflect their integration into the bacterial chromosome or co-selection by other antimicrobials still in use (Figure 3).

### 3.3 Genotypic profiles of *S.* Enteritidis isolates

**Figure 4:**
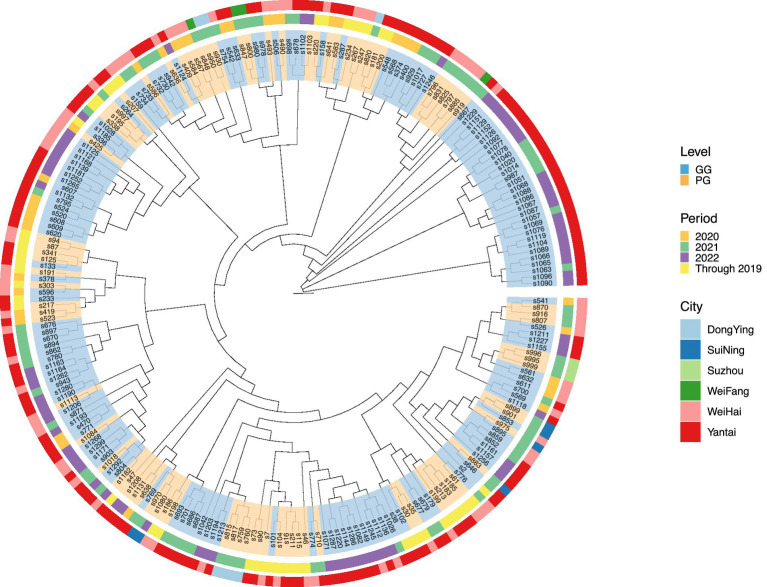
SNP tree analysis of *S.* Enteritidis isolates of breeder poultry company.

All 234 *S*. Enteritidis isolates were identified as multisite sequence typing (MLST) type 11. The phylogenetic tree was constructed based on the core genome sequence extracted from *S*. Enteritidis. Four concentric rings labeled the key attributes of each isolate: the innermost ring (Level) indicated the chicken house type (yellow = GG, blue = PG); the second ring (Year) indicated the year of isolation (2013–2022); the third ring (City) indicated the location of isolation. The study found that strains from the same city (e.g., Yantai, Weihai) mainly clustered within the same branch, indicating geographically specific transmission and limited inter-regional gene flow. Multiple branches containing both GG and PG isolates indicated vertical transmission of *S.* Enteritidis from ancestor to parent. The major branches included isolates spanning multiple years, indicating the long-term survival of specific *S.* Enteritidis clonal lineages in breeding farms. This temporal continuity highlights the challenges of eradicating *Salmonella* in intensive farming environments (Figure 4).

### 3.4 SNP homology analysis of *S.* Enteritidis

**Figure 5:**
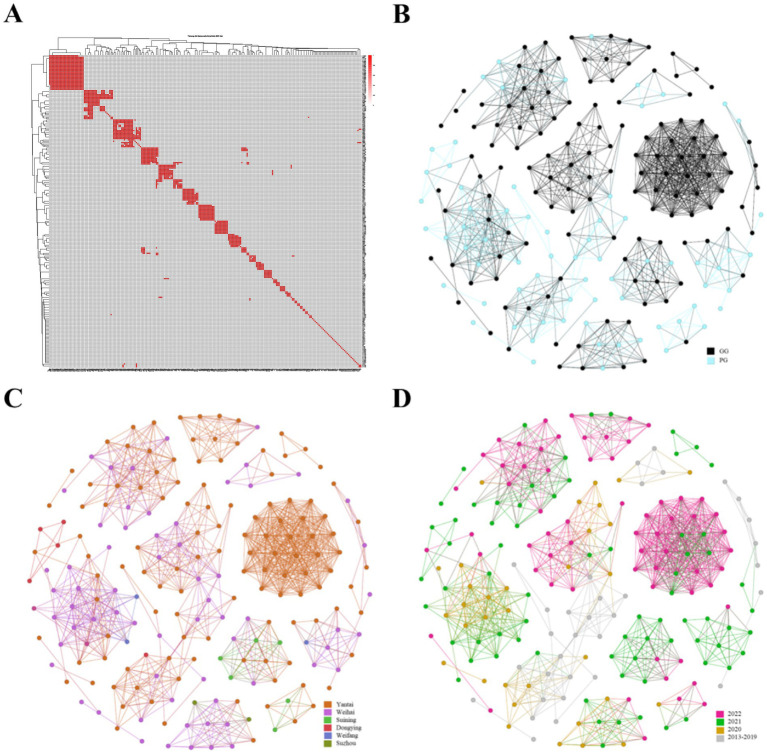
Analysis of clonal transmission of *S*. Enteritidis. **(A)** Clonal transmission map; **(B)** Transmission networks between grandparents and parents; **(C)** Transmission networks between different regions; **(D)** Transmission networks across different time periods.

Prokaryotic gene annotation (PROKKA) and pan-genome analysis (Roary) were used to extract the core genome and calculate SNP distances among the 234 *S.* Enteritidis strains. A strict international threshold of ≤5 core-genome SNPs was applied to define clonal transmission events (indicating clonal relatedness with minimal genetic divergence). Figure 5 Heatmap of SNP distance matrix among *S.* Enteritidis isolates. The heatmap visualizes pairwise SNP distances between strains, with red squares indicating clonal transmission events (SNP ≤ 5) and gray squares representing non-homologous relationships. Of the 234 strains, 163 (69.7%) showed homology to at least one other isolate, resulting in a total of 1,047 clonal transmission events (Figure 5A). Using Gephi, we constructed a network that demonstrated multiple clonal transmission events and highly similar genotypes among strains isolated from the same region (Figure 5B). Notably, the network also revealed extensive cross-transmission between distinct regions and between grandparents and parent generations (Figure 5C). Furthermore, temporal analysis suggests that *S.* Enteritidis strains have undergone widespread clonal transmission since 2013 (Figure 5D).

## 4 Discussion

The present study employed whole-genome sequencing (WGS) to conduct a decade-long longitudinal epidemiological investigation of *Salmonella* in a major integrated breeder poultry company in Shandong Province, China. By analyzing the serotypic distribution, genetic relatedness, transmission networks, and antimicrobial resistance (AMR) evolution of 252 *Salmonella* isolates, we obtained critical insights into the dynamics of *Salmonella* spread in the upstream segment of the poultry industry.

*Salmonella* infection remains one of the major public health concerns worldwide (26). This pathogen exhibits a prominent colonization advantage: once established in poultry farms, it can spread via both horizontal and vertical transmission. Notably, vertical transmission may lead to *Salmonella* contamination of eggs and successive chicken flocks, causing intergenerational amplification and thus the long-term persistence of *Salmonella* in poultry production systems (27). In traditional small-scale backyard poultry farming, a diverse range of *Salmonella* serovars have been isolated, primarily including *Salmonella Pullorum*, *Salmonella Gallinarum*, *S.* Enteritidis (SE), and *Salmonella Typhimurium* (28). Consistent with relevant studies, the results of this study revealed that with the development of intensive poultry farming, the prevalent *Salmonella* serovars have gradually become monomorphic, dominated by SE (27). A study by Jing *et al.* (29) on *Salmonella* in Shandong Province, China, from 2009 to 2012 also confirmed that SE was the most prevalent serovar during this period. Similarly, Yang et al. (30) found that SE was the predominant *Salmonella* serovar in large-scale breeder poultry enterprises. In addition, in line with the international consensus, all SE isolates obtained in this study belonged to sequence type 11 (ST11) (31).

Antimicrobial resistance (AMR) in chicken-derived *Salmonella* is largely attributed to the extensive use of antimicrobial agents (32), while mobile genetic elements (MGEs) such as plasmids and transposons play a central role in the acquisition and dissemination of antibiotic resistance genes (ARGs) (33). In this study, ARGs including *gyrA*, *bla_TEM-1B*, *aph*(3″)-*Ib*, *aph*(6)-*Id*, and *sul2* were commonly carried by isolates, indicating a heavy AMR burden in breeder chicken flocks. China fully implemented the ban on antibiotic growth promoters (AGPs) in animal feed in July 2020, prohibiting feed manufacturers from producing commercial feed containing AGP additives; in 2021, the country further promoted antimicrobial reduction initiatives for poultry and other livestock, setting a clear target of over 50% reduction in antimicrobial use in large-scale farms (34). Under this policy intervention, the present study found a significant decline in the carriage rate of the tetracycline resistance gene *tet*(A), plummeting from 72% in 2013–2020 to 26% in 2022. This change directly reflects the elimination of selective pressure for tetracyclines and confirms the effectiveness of targeted policy interventions in curbing the spread of plasmid-borne ARGs. In contrast, the presence rates of *gyrA*, *bla_TEM-1B*, and other core ARGs have remained high. It is possible that these ARGs have been integrated into the bacterial chromosome, thus exhibiting greater stability and being less prone to loss (35). Secondly, the use of therapeutic antibiotics may have maintained the continued presence of these genes in the Salmonella genome (36). Widespread AMR *Salmonella* in breeder chicken flocks can enter the food chain through contaminated poultry products, thereby limiting treatment options for severe human salmonellosis (37). The results of this study emphasize that even after the ban on AGPs in feed, continuous monitoring of feed and therapeutic antibiotic use is still necessary to prevent the unregulated use of therapeutic antibiotics from leading to the persistent prevalence of AMR.

SE accounted for as high as 92.85% of all isolates in the farms investigated in this study, occupying a dominant position. The transmission of SE has unique characteristics: in chicken flocks, it spreads not only horizontally but also vertically, with vertical transmission playing a pivotal role (38). The ovaries and oviducts of laying hens are the main colonization sites of SE, where the pathogen can survive for a long time and reproduce in large numbers, and then transmit vertically to the next generation of chicks through breeder eggs. This not only causes infection and morbidity in chicks and a reduced survival rate but also leads to the intergenerational transmission of *Salmonella* in chicken flocks; meanwhile, contaminated breeder eggs may also enter downstream production links, threatening the safety of poultry products (39). Phylogenetic analysis and core genome multilocus sequence typing (cgMLST) results of this study clearly showed that SE strains isolated from grandparent (GG) and parent (PG) flocks exhibited a mixed distribution pattern in the phylogenetic clades with extremely short genetic distances. This finding provides direct genomic evidence for the occurrence of vertical transmission between the two flocks. In addition, highly homologous SE strains were detected in farms in different regions, further confirming the important role of horizontal transmission in the spread of this pathogen. It is speculated that human mobility, cross-use of equipment, and feed contamination may be the main routes of horizontal transmission. At the same time, the persistent presence of SE in breeder farms over many years indicates that the current biosecurity measures implemented in breeder farms are still inadequate and insufficient to completely eliminate the colonization and transmission of this pathogen. Such persistent colonization may be associated with the ability of *Salmonella* to form biofilms for long-term survival at specific environmental sites (40) and to evade the host immune response (41). The underlying mechanisms still require further in-depth research to provide support for optimizing prevention and control strategies.

This study provides a genomic basis for formulating targeted *Salmonella* control strategies for the upstream links of the poultry production chain. Specifically, prevention and control efforts can be advanced from three aspects: first, given the absolutely dominant position of SE, it is necessary to implement serovar-specific monitoring protocols in breeder farms and adopt whole-genome sequencing (WGS) as the core technical means for high-resolution tracking of *Salmonella* transmission events; second, strengthen biosecurity measures in breeder farms, focusing on reducing the risks of vertical transmission and cross-regional spillover, while enhancing environmental cleaning and disinfection at hotspots of biofilm formation; third, the differential responses of ARGs to the AGP ban policy highlight the important link between antibiotics and AMR, thus it is essential to restrict antibiotic use and promote alternative antimicrobial strategies such as probiotics and phage therapy to reduce the selective pressure of antimicrobial agents while ensuring animal health. This study has certain limitations, which also point out the direction for future research: first, the study strains were only obtained from a single enterprise, resulting in limited generalizability of the research results; future studies need to expand the monitoring scope to cover multiple regions and enterprises to more comprehensively grasp the epidemiological characteristics of *Salmonella* in Chinese breeder poultry; second, combining genotypic resistance data with phenotypic antimicrobial susceptibility testing (AST) data can further improve the accuracy of AMR prediction; third, including environmental samples such as feed, litter, and drinking water as well as isolates from downstream commercial chicken flocks will help to fully elucidate the complete transmission chain of *Salmonella*. In conclusion, this decade-long genomic surveillance study clearly clarifies the core roles of vertical transmission and clonal expansion in the spread and AMR evolution of *Salmonella* in the breeder poultry system, which is of great significance for reducing the transmission of *Salmonella* in the poultry production chain, safeguarding the safety of poultry products, and protecting public health.

## Data Availability

The whole-genome sequencing data generated in this study has been deposited in the NCBI BioProject database under accession number PRJNA1440926.

## Footnotes

1
https://github.com/tseemann/snp-dists
